# Potential of Bovine Herpesvirus Vectors for Recombinant Vaccines

**DOI:** 10.3390/vaccines14010006

**Published:** 2025-12-20

**Authors:** Eda Mert Gokduman, Mustafa Ozan Atasoy, Ayşe Gencay Goksu, İbrahim Sozdutmaz, Muhammad Munir

**Affiliations:** 1 Division of Biomedical and Life Sciences, Faculty of Health and Medicine, Lancaster University, Lancaster LA1 4YQ, UK; e.mertgokduman@lancaster.ac.uk; 2 Department of Virology, Division of Preclinical Sciences, Faculty of Veterinary Medicine, Erciyes University, Kayseri 38030, Türkiye; agencay@erciyes.edu.tr (A.G.G.); isozdutmaz@erciyes.edu.tr (İ.S.); 3 Department of Veterinary Virology, Division of Preclinical Sciences, Faculty of Veterinary Medicine, Sivas Cumhuriyet University, Sivas 58140, Türkiye; mozan@cumhuriyet.edu.tr

**Keywords:** bovine herpesviruses, viral vaccine vector, CRISPR/Cas9, genetic engineering, codon optimization, codon de-optimization, bacterial artificial chromosomes (BACs)

## Abstract

The livestock industry experiences significant economic losses as a result of viral infections. Building on recent advances in biotechnological research, recombinant viral vector vaccines have emerged as promising platforms for next-generation vaccines. These vaccines can overcome many limitations of conventional vaccines, as they provide stronger protective immune profiles, stability, and improved safety profiles for various diseases. Bovine herpesviruses serve as viral vector platforms utilized due to their large genome capacity, potential for multigenic antigen delivery, and significant immune stimulation. In this review, we explored the structural characteristics and genomic organization of bovine alphaherpesviruses (BoHV-1, BoHV-4, and BoHV-5), covered BoHV-5 biology and attenuation strategies as part of the comparative platform analysis, and summarised the latest advancements in molecular tools used for viral genome editing. We further highlight the development of vaccines against bovine and zoonotic pathogens, discuss applications of BoHV-based vectors, and deliberate on future directions to improve vaccine efficacy. It also discussed the current state of research in the field, considered prospects, and outlined strategies for impending research. BoHV vectors are promising candidates as next-generation vaccine platforms in veterinary medicine and will play an important role in integrated disease control in livestock.

## 1. Introduction

The term “viral vector” refers to a broad group of viruses that can be engineered to deliver specific gene materials into cells for various purposes [1]. Recombinant vector vaccine systems are frequently employed to generate innovative vaccine candidates for viral infectious agents, which have a significant impact on the livestock industry. Various viral vector systems have been used to advance the development of recombinant vector vaccines. Non-replicating adenovirus vectors and the highly attenuated poxvirus vector Modified Vaccinia Ankara (MVA) may serve as promising vaccine platforms [2,3]. DNA plasmid vectors that express BoHV glycoproteins such as gB, gC, and gD may serve as potent DNA-based vaccines and offer safety and long-term stability [4].

Bovine herpesvirus 1 (BoHV-1), and Bovine herpesvirus 4 (BoHV-4) have been genetically engineered to act as viral vectors enabling heterologous antigen expression and delivery, thereby utilised for the development of multivalent vaccines targeting multiple bovine pathogens [5,6,7]. On the other hand, molecular approaches have been used to attenuate BoHV-5 through the knockout of non-essential genes, rather than through knock-in strategies to introduce heterologous genes for delivery purposes [8]. Vector vaccines can successfully stimulate humoral and cell-mediated immunity; however, their primary drawback is the immunological response elicited against the vectors themselves, which may reduce vaccine efficacy upon repeated administration [2]. Therefore, it is evident that there is a need for improved vaccine candidates to more effectively manage both acute and latent diseases in cattle populations. There is an increasing focus on techniques utilising gene-deleted or inserted vector vaccines to improve safety and facilitate serological differentiation [9,10]. Advanced genome editing approaches are being employed to generate viral vector vaccine candidates capable of inducing balanced humoral and cell-mediated immunity, reducing the spread and transmission of viruses, and preventing the establishment of latency.

The main objective of this review is to contextualize recent advances in the use of bovine herpesviruses as viral vectors for vaccine development in livestock. We sought to summarise the genomic organization and structural features of BoHVs, outline the main genetic engineering methodologies applied to generate BoHV-vectored vaccines, and evaluate their applications against various diseases. In addition, the review highlighted the key issues associated with these vector platforms and discussed future perspectives for improving vaccine efficacy and safety in cattle. While doing so, we mainly highlighted the most prominent bovine herpesviruses, BoHV-1, BoHV-4, and BoHV-5, all of which are members of the *Orthoherpesviridae* family, although they vary in subfamily classification.

## 2. Genomic Structure and Organization of BoHVs

**Figure 1:**
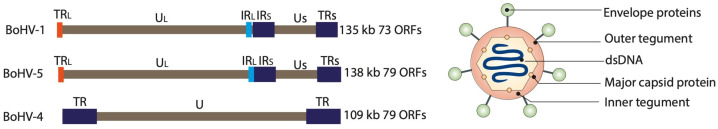
Genomic structure of Bovine herpesviruses. U: Unique region, UL: Unique long region, US: Unique short region, TR: Terminal repeat, TRL: Terminal repeat long, TRS: Terminal repeat short, IRL: Internal repeat long, IRS: Internal repeat short, ORF: Open Reading Frames, dsDNA: double stranded DNA.

BoHV-1 and BoHV-5 belong to the *Alphaherpesvirinae* subfamily and share a high degree of genomic and structural similarity, whereas BoHV-4 is classified within *Gammaherpesvirinae* subfamily [11,12]. These viruses exhibit the classical herpesvirus architecture but differ in their genome organization (Figure 1). The virion structure of bovine herpesviruses consists of a double-stranded DNA (dsDNA) core enclosed within an icosahedral capsid, surrounded by a tegument layer, and enclosed by an outer envelope studded with glycoproteins [13].

BoHV-1 and BoHV-5 possess a D-type herpesvirus genome arrangement consisting of a unique long (UL) region and a unique short (US) region. These regions are flanked by internal repeat (IR) and terminal repeat (TR) sequences, and viral genes are distributed across the UL, US, and repeat regions [14,15]. The BoHV-1 genome is approximately 135 kb in length and encodes around 72 open reading frames (ORFs) [16]. Similarly, the BoHV-5 genome is about 138 kb in length and contains approximately 79 ORFs, most of which are orthologous to BoHV-1 genes [17]. Many essential viral genes required for virus replication, structural assembly, and immune evasion, are located within the UL region, whereas the US region encodes several envelope and accessory proteins homologous to those found in other alphaherpesvirus [18]. In contrast, BoHV-4 has a B-type herpesvirus genome arrangement and exhibits a different genomic organization, with a length of approximately 144 kb (Figure 1). The BoHV-4 genome has a unique region (U) flanked by multiple terminal repeat (TR) sequences and encodes 79 ORFs, including genes involved in latency, immune modulation, and host-cell interaction—these are characteristic features of gammaherpesviruses [15].

## 3. A Comparative Analysis of BoHVs

An initial systematic comparison of BoHV-1, BoHV-4, and BoHV-5 is essential for assessing their relative suitability as viral vectors. Although these three herpesvirus species share several genomic and biological features, they differ markedly in genome plasticity, tissue tropism, latency patterns, pathogenicity, pre-existing immunity, and their capacity to induce systemic and/or mucosal immune responses. These core characteristics directly influence the safety and immunogenic potential of the vector candidates.

BoHV-1 replicates in the respiratory epithelium and establishes lifelong latency in sensory neurons, primarily within the trigeminal ganglion [19]. Its neurotropic latent infection raises significant biosafety concerns when utilised as a live vector, particularly with respect to potential latency reactivation or genomic recombination, even when attenuating deletions such as gE or TK are introduced [7]. Nevertheless, BoHV-1’s respiratory tropism may offer advantages for vaccines targeting pathogens that require strong mucosal immunity, as it can enhance local antigen presentation in the upper respiratory tract. Despite evidence of both humoral and cellular immune responses, quantitative measures of mucosal immunity remain inconsistently reported across studies [9,20].

BoHV-4 exhibits a significantly distinct biological profile. It has a wide range of in vitro cell tropism, predominantly remains inactive in lymphoid tissues, and circulating mononuclear cells [21]. This type of lymphotropic latency mitigates the risk of neurological reactivation commonly associated with other alphaherpesviruses. Additionally, it may facilitate the long-term retention of antigens in the body without causing neuronal damage. BoHV-4 usually exhibits lower seroprevalence across cattle populations [22], which offers a strategic advantage, as pre-existing immunity could dramatically reduce vector efficacy. Furthermore, BoHV-4 possesses a relatively longer genome; an extensive long unique region (LUR) enables the insertion of heterologous sequences and the generation of replication-deficient viral vectors by targeting essential genes. BoHV-4-vectored vaccines have demonstrated antigen-specific systemic and mucosal responses following intranasal (IN) administration; however, quantitative correlates remain limited and require further standardised evaluation [9]. BoHV-5, genetically related to BoHV-1, is characterised by severe neurovirulence and a strong tropism for the central nervous system, where it is implicated in causing meningoencephalitis [7]. This virus species greatly limits its suitability as a live vector, unless extensive attenuation or replication-deficient methods are utilised.

Although BoHV-5 shares a genome size and overall structure similar to BoHV-1, it exhibits a greater propensity to cause neurological disease. Consequently, strict safety assessments are required, including evaluation of neuroinvasion risk as well as studies on latency and reactivation. In principle, BoHV-5 has vector capabilities comparable to BoHV-1; however, scarcity of published preclinical vector research and its high neurovirulence necessitates thorough risk assessment prior to practical application. Altogether, it is plausible to hypothesise that selecting an appropriate herpesviral vector platform depends critically on the nature of the targeted pathogen and the desired immune response, while ensuring a high level of safety. For mucosal pathogens (e.g., BRSV, BVDV, FMDV), vectors capable of eliciting strong IgA and tissue-resident T-cell responses in the upper respiratory tract may be preferred. Conversely, systemic humoral immunity may be better achieved using platforms with low pre-existing immunity and minimal safety risks.

## 4. Methodologies to Engineer BoHV-Vectored Vaccines

### 4.1. Homologous Recombination

Homologous recombination (HR) is a natural genetic process in which nucleotide sequences are exchanged between similar DNA molecules. In double-stranded DNA viruses, HR represents one of the oldest and most fundamental strategies of genetic manipulation [23]. This intrinsic mechanism enables the introduction of defined modifications into viral genomes. For the development of viral vector vaccines, a donor plasmid containing the foreign gene(s) flanked by sequences homologous to target region of viral genome is constructed. Homologous recombination occurs when the viral genome and the donor plasmid exchange matching DNA regions, allowing the foreign gene to be transferred into the virus. When both the viral genome and the plasmid are co-transfected into host cells, this recombination takes place at these matching sites, resulting in a recombinant virus that carries the inserted gene [24]. Engineered herpesvirus vectors generated through homologous recombination have been employed in vaccine development to deliver antigens from various pathogens, including foot and mouth disease virus (FMDV) [25], bovine viral diarrhea virus (BVDV) [24], and Rift Valley fever virus (RVFV) [26]. HR is often integrated with other genome manipulation technologies, such as bacterial artificial chromosome (BAC) systems [27] and CRISPR/Cas9-based approaches [28], to facilitate efficient manipulation and recovery of viral genomes.

### 4.2. Bacterial Artificial Chromosomes (BACs)

Bacterial artificial chromosome (BAC) technology has enabled the manipulation of large DNA molecules, including most of the herpesviral genomes [29]. BACs are artificial DNA constructs derived from the F-plasmid and are designed to clone large DNA fragments in *Escherichia coli* (*E. coli*). Using this system, an entire viral genome can be transferred into a BAC plasmid in infected cells. After insertion, the circularised BAC–viral DNA hybrid is isolated from cells and transformed into competent *E. coli*. Then, the BAC DNA can be transfected back into permissive eukaryotic cells. The BAC DNA can then be transfected back into permissive eukaryotic cells, where it reconstitutes infectious recombinant viruses and produces vectors expressing foreign genes for vaccine development [30]. The ability to modify viral genomes by inserting or deleting sequences makes a BAC system potent and efficient tool to deliver numerous genes from various infectious agents. For instance, the BoHV-4-BAC viral vector was engineered to express a truncated glycoprotein D of BoHV-1 to assess its immunogenicity in the BALB/c mouse model [31]. This vector was compared with a DNA vaccine expressing the same antigen. Following immunisation, both vaccines elicited strong immune responses, and the BoHV-4 vector showed promise as an alternative to the DNA vaccine. Similarly, BoHV-4 has been reconstructed using the BAC system to generate vector for several vaccine candidates, including BVDV [6], Bluetongue Virus [32], and zoonotic pathogens such as Crimean-Congo hemorrhagic fever virus [33] and monkeypox virus [34]. BAC-based genomic clones have also been developed for BoHV-1 and BoHV-5 [30], but they are relatively less common for recombinant vector applications compared with BoHV-4 (see Table 1).

### 4.3. Codon Optimization and Codon De-Optimization

Codon optimization of target genes has been widely used in the generation of recombinant viral vectors to improve recombinant protein expression in different hosts, such as mammalian cells or yeasts [13]. This approach aims to enhance heterologous protein expression levels by designing the target gene sequences that match the host’s preferred codon usage [35]. In BoHV vector development, codon optimization is applied to antigen-encoding genes to improve protein expression in mammalian cells and increase mRNA stability. Previous studies indicated that codon optimization can elevate viral protein expression, maintain viral replication competence, and increase antigen yields in genetically stable recombinant herpesvirus-based vaccines [24,28].

**Figure 2:**
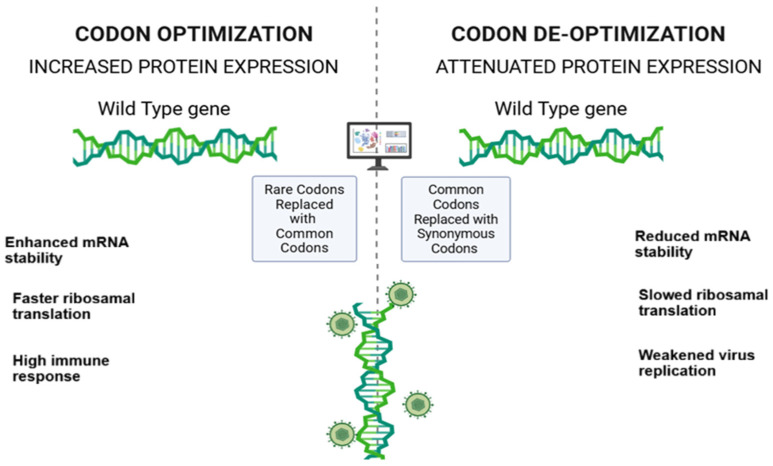
**Approaches to develop vaccines.** Comparison of codon optimization and codon de-optimization in viral vector vaccine development. Codon optimization enhances antigen production for subunit or inactivated vaccines, while codon de-optimization creates attenuated live vaccines by weakening viral replication, both critical strategies in advanced veterinary vaccine development.

Codon deoptimization, on the other hand, is a reverse strategy designed to reduce translation efficiency by recoding viral genes with host-rare or suboptimal codons. This approach has been applied in the development of live-attenuated vaccine candidates [36] (Figure 2). Codon pair bias deoptimization (CPBD) and codon usage deoptimization (CUD) are key strategies that attenuate viruses by altering target genetic sequences to lower translation efficiency, thereby reducing replication capacity and pathogenicity [37]. CUD replaces commonly used codons with less frequent synonymous codons, forcing the virus to rely on scarce host tRNAs and consequently slowing replication and decreasing viral fitness. In contrast, CPBD alters the frequency of specific codon pairs based on patterns observed in natural genomes. Overall, codon deoptimization preserves the antigenic profile of the virus, maintaining strong immunogenicity while reducing pathogenicity [38,39]. This strategy supports the development of safer vaccines with reduced risk of reversion. Although largely explored in RNA viruses such as poliovirus, influenza A, and dengue virus, recent research has extended its use to large double-stranded DNA viruses, including herpesviruses [37,40,41]. The oncogenic Marek’s disease virus (MDV) provided the first demonstration of codon deoptimization attenuation in a herpesvirus by recoding the UL30 gene, which encodes the catalytic subunit of the DNA polymerase. Partial deoptimization of UL30 led to significantly decreased protein synthesis, impaired replication kinetics, and reduced pathogenicity in vivo [40]. Furthermore, CUD and CPBD have been applied to infectious laryngotracheitis virus (ILTV). Experimental evidence showed that CPBD could effectively attenuate ILTV while preserving viral viability, whereas extensive CUD might be overly disruptive and can sometimes prevent the recovery of viable virus. [37].

To date, CUD and CPBD strategies have not yet been experimentally applied to BoHVs; therefore, their potential use in these viruses remains speculative. Nevertheless, existing studies indicate that codon deoptimization could be applied to develop live-attenuated vaccine candidates for other large DNA viruses, such as bovine herpesviruses, particularly when used in combination with additional genetic engineering tools. Integrating codon optimisation tools with advanced genome editing systems such as BAC recombination and CRISPR/Cas9 could support the creation of genetically stable, high-yield recombinant viral vectors that provide robust platforms for vaccine development and functional virology research.

### 4.4. The Clustered Regularly Interspaced Palindromic Repeats and CRISPR-Associated Systems (CRISPR-Cas9)

**Figure 3:**
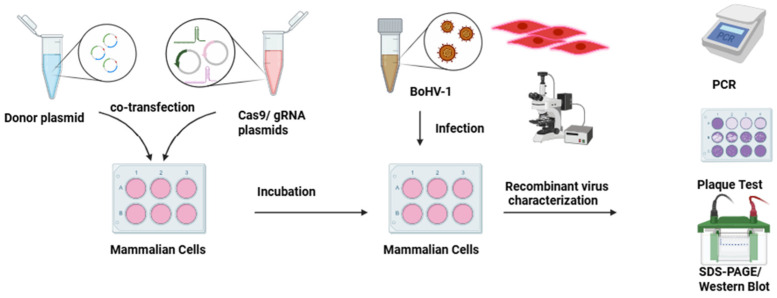
General experiment design for BoHV genome editing using the CRISPR/Cas9 technique. The antigenic target gene is cloned in the donor plasmid. Then, donor plasmids and gRNA/Cas9 plasmids are co-transfected into cells. Thereafter, transfected cells are infected with BoHV. Recombinant viruses are followed by fluorescent tags and are characterized by plaque tests, PCR, and Western blot analyses.

The clustered regularly interspaced palindromic repeats (CRISPR) and CRISPR-associated systems (Cas) have been extensively developed and applied to enable accurate and rapid gene editing (Figure 3). The CRISPR/Cas9 system allows efficient insertion or deletion of genes in viral genomes [28]. This technique functions by generating site-specific double-stranded DNA breaks that trigger homology-directed repair (HDR) or non-homologous end joining (NHEJ), thereby improving the precision and speed of target sequence modification within viral genomes [42]. These repair mechanisms support robust transgene expression, increase genetic stability, and facilitate efficient production of recombinant viruses [43]. The CRISPR/Cas9 method has recently been applied to bovine herpesviruses for several purposes, including elucidating viral gene functions [44], deleting virulence genes to attenuate the virus [28], and generating vector vaccine candidates [10,45,46]. Combining CRISPR/Cas9 with homologous recombination has enabled the production of attenuated recombinant BoHV-1 viruses through deletion of the glycoprotein E (gE) gene, demonstrating a successful strategy for viral gene editing with clear applications in vaccine development [20]. Another CRISPR/Cas9 mediated recombinant BoHV-1 was produced by replacing the gE gene with the rabies virus glycoprotein (RABVG), which effectively expresses the RABV G protein without altering the parental BoHV-1’s growth characteristics. This modification led to stable expression of RABVG after multiple passages in cells and induced neutralising antibodies (NAs) against rabies in both mice and cattle [10].

Yu et al. (2024) [46] have developed a CRISPR/Cas9–HDR platform to construct a recombinant BoHV-1 virus with gI and gE deletions and insertion of a fluorescent marker gene and further evaluated the biological characteristics of this emerging strain. Their results showed that the mutant virus exhibited growth kinetics comparable to the parental strain but demonstrated a high level of attenuation while retaining immunogenicity, as evidenced by comparable gB-specific antibody responses in the mouse model. Their platform simplifies and accelerates the construction of BoHV-1 mutants [46]. Taken together, these studies demonstrate that CRISPR/Cas9 technology can overcome key technical difficulties in BoHV-1 genome editing and streamline the development of vector-based vaccines for viral infections.

## 5. Bovine Herpesvirus-Based Platforms for Multivalent and Cross-Species Vaccine Development

### 5.1. Bovine Respiratory Syncytial Virus (BRSV)

BRSV is an enveloped RNA virus of the *Pneumoviridae* family that causes severe respiratory disease in calves. The BRSV glycoprotein G antigen gene was inserted into BoHV-1 by homologous recombination and evaluated in calves. An early study by Schrijver et al. (1997) constructed a G-gene knock-in, gE-deleted BoHV-1 and evaluated it against virulent BRSV and BoHV-1 in SPF calves [47]. Two-dose administration via intranasal and intratracheal routes of the bivalent vaccine candidate conferred protection against wild-type BRSV, inducing a 1000-fold increase in neutralising antibody titres and reducing viral shedding beginning at 5 days post-challenge (dpc). Furthermore, the vaccine candidate reduced viral shedding titres of wild type BoHV-1 by approximately 100-fold and shortened the duration of shedding to ~7.3 days. These findings suggest that BoHV-1 can efficiently present respiratory antigens.

### 5.2. Bovine Viral Diarrhea Virus (BVDV)

BVDV causes respiratory, enteric, and reproductive disorders in cattle and leads to substantial economic losses in the livestock industry. BoHV-1-based vectors encoding the BVDV envelope glycoprotein E2 were developed using homologous recombination and CRISPR/Cas9-mediated techniques. Studies reported strong antigen-specific and neutralising antibody responses in both experimental and natural hosts like guinea pigs, mice, and calves (Table 1) [45,48,49]. A recent study by Liu et al. (2022) introduced a chimeric BVDV E2 protein bearing the gD signal peptide into the gE region of BoHV-1 [45]. The recombinant virus was evaluated in calves challenged with both wild-type viruses. The bivalent vaccine candidate induced BoHV-1–specific neutralising antibody titres comparable to those observed in the vector control (387.63 and 337.59 SN_50_, respectively). In contrast, BVDV-specific neutralising antibody titres were significantly lower (21.82 SN_50_) than those induced by wild-type BVDV (337.59 SN_50_); nevertheless, the vaccine candidate conferred strong protection based on clinical and pathological findings. Similarly, recombinant BoHV-4 mutants have been constructed for expression of BoHV-1 glycoprotein D and BVDV E2 antigens. These recombinant BoHV-4 viruses induced strong humoral immune responses in rabbits and cattle [5,9,50]. For instance, a similar chimeric E2–gD construct incorporating a signal peptide was introduced into BoHV-4 by Donofrio et al. [50], whose findings showed that rabbits immunised with the recombinant strain developed BVDV-neutralising antibodies that increased from undetectable levels to approximately log_2_ 3 (1:8 neutralization-assay endpoint) at week 2 and reached a plateau of log_2_ 5–6 by early week 3. The vaccine also elicited BoHV-1 gD-specific ELISA antibodies (OD ~0.25 at week 4); however, no BoHV-1-neutralising antibodies were detected. Altogether, these studies demonstrate that BoHV-4 has a high capacity for multigenic insertion and highlight its potential as a dual or multivalent vaccine vector. Overall, both BoHV-1 and BoHV-4 represent efficient and genetically stable platforms for the expression of pestiviral antigens.

### 5.3. Foot and Mouth Disease Virus (FMDV)

FMDV is a highly contagious *Aphthovirus* affecting cloven-hoofed animals, characterised by vesicular lesions and rapid viral spread [51]. BoHV-1 recombinant vectors constructed to express the FMDV VP1 gene or epitope induced variable immune responses depending on the animal model. An early endeavour by Kit et al. (1991) [23] introduced VP1 epitope gene into N terminus of gE position in TK deleted BoHV-1 mutant. In vivo trials indicated that calves vaccinated with the recombinant virus developed BoHV-1 neutralising titers of 4–32, strong anti-FMDV ELISA responses (peak OD or log values 1.75–2.88), and protective FMDV neutralising titers between 1:200 and 1:500, while anti-FMDV peptide antibodies selectively neutralised recombinant but not parental virus. They also reported that all vaccinated calves were fully protected from BoHV-1 challenge. Ren et al. (2009) further showed that their gE-deleted BHV-1/VP1 recombinant induced sufficient BoHV-1 neutralising antibodies after a single dose (rising from <2 to 1:8–1:16), while a booster was required to elevate titers up to 1:32 and to generate detectable VP1-specific ELISA responses, increasing from OD ~0.05 to ~0.20–0.25, demonstrating functional VP1 expression and a booster-dependent humoral response [25]. These differences underline the importance of host-specific vector performance and antigen presentation efficiency for BoHV-1.

### 5.4. Peste Des Petits Ruminants Virus (PPRV)

PPRV is a morbillivirus that causes a highly contagious disease in small ruminants, leading to significant respiratory and gastrointestinal symptoms. Considering the importance of disease in livestock industry, an initial attempt by Osman et al. (2018) involved integrating PPRV antigenic genes into the terminal region of gB; however, this resulted in a replication-deficient progeny virus that did not progress to in vivo evaluation [52]. Later, an in vivo replication-deficient BoHV-4 vector expressing the PPRV hemagglutinin protein (BoHV-4-A-PPRV-H-ΔTK) was constructed by Macchi et al. (2024) by using BAC recombination [53]. The constructed recombinant vaccine was administered to Harlan mice intraperitoneally with a single booster dose at 21 dpv. The results showed significant activation of CD4^+^ and CD8^+^ T cells, with CD4^+^ IFN-γ^+^ frequencies increasing by approximately 2.8-fold and CD8^+^ IFN-γ^+^ frequencies by ~3.3-fold following stimulation with inactivated PPRV. In parallel, strong PPRV-neutralising antibody titres ranging from 1:120 to 1:360 were detected seven days after booster vaccination, representing a 12–36-fold increase compared with the titres in control animals (<1:10). Depending on these promising findings, the vector was further evaluated in the natural host, confirming its protective efficacy against the virulent PPRV challenge. Sheep were vaccinated at the same intervals by intramuscular injection and subsequently challenged with the virulent ICV’89 PPRV strain. The results demonstrated an approximately 45-fold increase in IFN-γ responses to the H9 peptide of PPRV, following stimulation with BEI-inactivated PPRV. Flow-cytometry further confirmed the early expansion of CD4^+^ and CD8^+^ T-cell populations, which increased by approximately 1.7- to 1.9-fold between days 2 and 7 post-challenge. In addition, vaccinated animals developed robust neutralising antibody titres, representing an 8- to 16-fold increase compared with the control group. Neither viremia nor viral shedding was detected in vaccinated animals, indicating a reliable sterilising protection in sheep [21].

### 5.5. Rabies Virus (RABV)

The rabies virus is a neurotropic *Lyssavirus* that causes fatal encephalitis in mammals, including humans and livestock [54]. CRISPR/Cas9 and homologous recombination techniques were combined to generate BoHV-1 expressing the rabies glycoprotein G (RABVG). The RABVG gene was inserted into the BoHV-1 genome between the gI and US9 genes, resulting in stable expression of the rabies glycoprotein without affecting the viral growth kinetics. In vivo analysis of the recombinant BoHV-1 in mice and cattle demonstrated that the vaccine generated strong immune responses and induced higher titres of virus-neutralising antibodies against rabies. Zhao et al. (2022) showed that a single IM dose of the gE-deleted BHV-1 recombinant expressing RABVG induced strong rabies virus–neutralising antibody levels in mice and cattle, with VNA titres exceeding the protective threshold of 0.5 IU/mL and reaching ~1–3 IU/mL in mice (persisting for 8 weeks) and 0.8–2.0 IU/mL in 6–8 months old cattle (weeks 1–12), while also activating cellular immune response significantly more than control groups [10]. The study illustrated that BoHV-1 is a safe and effective platform for expressing foreign viral antigens, a capability further strengthened by precise genome-editing methods.

### 5.6. Rift Valley Fever Virus (RVFV)

RVFV is a mosquito-borne *Phlebovirus* causing severe disease in ruminants and is a major etiological agent of abortion storms in livestock herds [55]. Recent complementary studies by Palvuraj et al. have provided compelling evidence that herpesviral vectors possess substantial versatility and utility as platforms for the development of vaccine candidates targeting pathogens of zoonotic concern. They constructed a recombinant BoHV-1 deleting four genes (UL49.5, glycoprotein G (gG), gE cytoplasmic tail (gECT), and US9), resulting in a vector termed BoHV-1qmv, engineered to express chimeric RVFV Gn-GMCSF (Granulocyte-macrophage colony-stimulating factor) or Gc proteins [26]. Later, they characterized the immunogenicity of the constructed virus using quantitative serological analyses in both sheep and calves [26,56]. Sheep vaccinated intranasally (IN) and subcutaneously elicited a strong humoral immune response against BoHV-1, which peaked around 21 days post-primary vaccination (approximately a 60-fold increase). The prime-boost administration on day 29 further increased the SN titre to 1:128, indicating that the vector retains sufficient immunogenicity after genetic modification. RVFV-specific neutralising antibodies were also evaluated, showing detectable titres at 7 dpv that reached their maximum at day 21 (a 26-fold increase) and further rose to 1:64 after boosting. Cellular immunity, assessed by antigen-stimulated PBMC responses, was also robust, showing up to a 4.85-fold increase in IFN-γ expression slightly higher than the attenuated RVFV vaccine (MP-12, 3.5-fold) [56]. In 6-month-old calves, the BoHV-1-specific humoral response developed similarly to that observed in sheep but at a lower magnitude, reaching a 12-fold increase at 7 dpv and peaking at a 36-fold increase by 21 dpi. The RVFV-specific neutralizing antibody response was detectable by 7 dpv (approximately 6.5-fold) and increased to a 17-fold rise by 21 dpv [26]. Cellular immunity, assessed using the same approach by quantifying IFN-γ transcripts via real-time PCR, demonstrated vaccine-induced cell-mediated immune activation, showing an 8.34-fold increase in interferon-γ expression.

The quadruple gene deletion significantly improved the safety profile of the viral vector, as demonstrated by the absence of clinical disease and reduced virulence observed in immunised calves. This directly addresses key biosafety concerns associated with herpesvirus-based vectors. The vaccine virus replicated efficiently in the nasal epithelium, shedding live virus for at least five days post-immunisation; however, importantly, the BoHV-1qmv Sub-RVFV exhibited no shedding following dexamethasone-induced latency reactivation, indicating a substantially reduced risk of reactivation-mediated virus dissemination [56,57]. This improvement is critical for mitigating the risk of vaccine virus transmission to unvaccinated or immunocompromised animals, a common concern with live recombinant vaccines. Despite these deletions, the BoHV-1qmv Sub-RVFV maintained genetic stability with consistent RVFV antigen expression across serial passages. İmmunisation produced strong humoral and cellular immune responses specific to RVFV, such as neutralising antibodies and interferon-gamma production, even after a single dose. The incorporation of multiple gene deletions, including a serological marker gene, enables DIVA capability, which is important for disease surveillance. The quadruple gene deletion strategy not only reduces virulence and regulates nasal shedding dynamics but also enhances safety by restricting vaccine virus transmission. Consequently, this approach strengthens the utility of BoHV-1 as an effective and safe viral-vectored vaccine platform for cattle. Taken together, these findings indicate that BoHV-1 is a stable and efficacious platform for expressing heterologous antigens, capable of inducing immune responses without causing disease, while also demonstrating species-dependent variability yet confirming its applicability for arboviral antigen delivery in large animals.

### 5.7. Nipah Virus

Nipah virus is a bat-associated zoonotic *paramyxovirus* that possesses significant pandemic potential [58]. Both the envelope glycoprotein (G) and fusion protein (F) are essential for viral entry; G mediates receptor attachment, whereas F promotes pH-independent fusion of the viral envelope with the host cell membrane [59]. Thus, the two genes were individually incorporated into a BoHV-4 vector, and their efficiencies were comparatively assessed against a canarypox vector (ALVAC) in a porcine model by Pedrera et al. (2022) [60]. Serum neutralizing antibody titres in pigs immunized intramuscularly with two doses administered at 21-day intervals revealed that, although the G-expressing BoHV-4 mutant induced higher levels of neutralizing antibodies, the F-expressing virus exhibited superior neutralising capacity. Furthermore, IFN-γ ELISpot and flow cytometry analyses showed that BoHV-4-G elicited stronger antigen-specific CD4^+^ and CD8^+^ T-cell responses than ALVAC-G.

### 5.8. Viral Haemorrhagic Fever Viruses: Crimean-Congo Haemorrhagic Fever Virus (CCHFV) and Ebola Virus (EBOV)

CCHFV is a tick-associated virus classified within the Nairoviridae family and causes severe hemorrhagic fever in humans while remaining asymptomatic in animals, while contributing to endemic cycle of virus [61]. The broad host spectrum of BoHV-4 and the lack of pre-existing immunity in humans make it a considerable alternative platform for development of vectored vaccines against zoonotic threats. A study conducted by Farzani et al., (2019) introduced nucleoprotein gene of CCHFV into TK deficient BoHV-4 vector (BoHV4-ΔTK-CCHFV-N) through BAC system and challenged the efficacy, along with Ad5 DNA vector in ﻿FNα/β/γR−/− mice [33]. Comparative *immunological analyses revealed that* BoHV4-ΔTK-CCHFV-N induced a dominant IgG2b response (0.8–1.0 OD_450_ on day 28), indicating Th2-skewed humoral immunity, as well as a high level of N-specific total IgG production (1.2–1.4), but with non-neutralising characteristics. In addition, a moderate level of IFN-γ induction and robust proinflammatory cytokine responses (IL-6, IL-10, and TNF-α) were detected in mice. The lethal challenge test against a virulent CCHFV strain further revealed that the challenged groups presented clinical manifestations; however, the survival rate was 100% in the BoHV4-ΔTK-CCHFV-N group, and the animals recovered by day 10. As a result, the construct demonstrated a strong capacity to induce T cell–mediated immune responses, along with comparable protection in the lethal challenge study, suggesting that BoHV-4 represents a promising vaccine vector for zoonotic viruses.

Ebola virus disease (EVD) is a highly contagious and severe illness with high mortality caused by the Ebola virus, a member of the *Filoviridae* family [62]. Ebola virus entry into host cells is driven by interactions between the viral glycoprotein (GP) and host cell surface receptors; therefore, GP is often regarded as a major determinant of viral pathogenicity [63,64]. Given the critical functional importance of GP, Rosamilia and colleagues (2016) utilised BoHV-4 for a recombinant vector vaccine candidate [65], whose efficacy was evaluated in a goat model. After demonstrating the stability of the recombinant virus, they administered two SC doses at a two-week interval and measured Ig levels by ELISA, demonstrating that the virus conferred a robust and long-lasting antibody response lasting up to six months.

### 5.9. SARS-CoV-2

The emergence of the coronavirus pandemic in 2019 imposed substantial health, economic, and social burdens worldwide, prompting intensified virological research and the rapid development of vaccines [66]. A wide range of vaccine strategies has been conceptualised, validated, and implemented to date. In this regard, various human and animal herpesviruses, including herpes simplex virus as well as human and murine cytomegaloviruses, have been utilised as vectors for the delivery of the SARS spike protein [67,68,69,70]. An illustrative study by Park et al. (2023) developed a thymidine kinase–deficient BoHV-4–based vaccine expressing the SARS-CoV-2 spike protein using a BAC system and evaluated it in mice and Syrian hamsters following two-dose intranasal and intramuscular administration [71]. Vaccinated mice showed markedly increased neutralizing antibody titres against Wuhan, Alpha, Beta, and Gamma variants, with reduced activity against Omicron. Furthermore, cytotoxicity assays revealed a dose-independent, cellular immunity–mediated response directed against the spike glycoprotein in vaccinated animals. After challenge with the Wuhan strain of SARS-CoV-2, the vaccine candidate showed strong protection, as indicated by reduced weight loss and milder lung and tracheal pathology in hamsters.

### 5.10. Monkeypox Virus (MPXV)

Monkeypox has re-emerged as a significant global public health issue since the first alerts were issued in the United Kingdom, accounting for 137,892 confirmed cases worldwide from 1 January 2022 to 31 March 2025 [72]. Currently, live attenuated vaccines such as MVA-BN (Imvamune^®^, Bavarian Nordic A/S, Hellerup, Denmark) and LC16m8 (LC16-KMB^®^, KM Biologics, Kumamoto, Japan) have been approved. An experimental study by Franceschi et al. (2015), several immunogenic glycoproteins of MPXV, including A29L, M1R, and B6R, were selected to construct BoHV-4 recombinant viruses, and evaluated in vitro and in vivo [34]. The results showed that the BoHV-4 vector stably expressed MPXV proteins; however, B6R exhibited significantly lower viral kinetics, due to substantial cytotoxic effects in cell culture. In a lethal challenge using the STAT1^−^/^−^ mouse model, the results indicated that the BoHV-4–M1R recombinant conferred complete protection; however, it was associated with significant weight loss in mice compared with the MVA vaccine.

**Table 1:** Summary of experimental studies employing BoHV-1, BoHV-4, and BoHV-5 as vaccine or gene delivery vectors.

Vector	Target Disease	Antigen	Vaccine Construction Method	Animal Model	Observed Immune Responses	Ref.
BoHV-1	BRSV	Glycoprotein G (gG)	HR	Calves	Humoral response with high Ab titres	[47]
BoHV-1	BRSV	gG	HR	Calves	Humoral immune response induced partial protection	[73]
BoHV-1	BVDV	Envelope protein E2	HR	Guinea pigs and calves	Protection with high Ab titres	[48]
BoHV-1	BVDV	Envelope protein E2	RE-based	Mouse and calves	Humoral immune response with high Ab titres	[49]
BoHV-1	BVDV	Envelope protein E2	HR	Calves	High neutralising Ab titre	[24]
BoHV-1	BVDV	Envelope protein E2	CRISPR/Cas9	Guinea pigs and calves	High neutralising Ab titre	[45]
BoHV-1	FMDV	VP1 epitope	HR	Calves	Protective antibody	[23]
BoHV-1	FMDV	VP1	HR	Rabbits	Low immune response	[25]
BoHV-1	Rabies virus	gG	CRISPR-Cas9 and HR	Cattle and mice	Strong neutralising antibody	[10]
BoHV-1	RVFV	Glycoproteins Gn and Gc	HR	Calves	High antibody titre	[26]
BoHV-1	RVFV	Glycoproteins Gn and Gc	HR	Sheep	High antibody titres	[56]
BoHV-4	BoHV-1	gD	BAC	Rabbit	Elicited humoral immune response	[5]
BoHV-4	BoHV-1	gD	HR	Mice	High humoral immune response	[31]
BoHV-4	BoHV-1 and BVDV	gD (BoHV-1) gE2 (BVDV)	BAC	Rabbit	High humoral immune response	[50]
BoHV-4	BoHV-1 & BVDV	BoHV-1 gD + BVDV gE2	HR and BAC	Cattle	Antigen-specific neutralising Ab induction	[9]
BoHV-4	PPRV	Hemagglutinin glycoprotein	HR	Mice	High neutralising Ab titres	[53]
BoHV-4	Malignant catarrhal fever virus	Glycoprotein B	BAC	Rabbit	Modest neutralising Ab level, Partial protection (43% survival rate)	[74]
BoHV-4	PPRV	Hemagglutinin glycoprotein	HR	Sheep	Strong protection with high Ab titers	[21]
BoHV-4	CCHFV	Nucleocapsid protein	HR	Mice	Strong humoral responses	[33]
BoHV-4	Ebola	Glycoprotein	BAC	Goat	Long-lasting immunoglobulins	[65]
BoHV-4	Nipah	Fusion (F) and Glycoprotein (G) genes	BAC	Pig	Higher Ab titres and robust cellular immune response for G, Higher neutralising capacity of Ab for F,	[60]
BoHV-5	BoHV-5	gE and TK deletions	HR	Rabbit	High (gE), Low (TK)	[75]
BoHV-5	BoHV-1 and BoHV-5	gE and TK deletions	HR	Calves	Strong virus-neutralising Ab titre	[76]
BoHV-5	BoHV-5	gE, gI, and US9 deletions	HR	Calves	Strong neutralising Ab	[8]

## 6. Key Challenges in Bovine Herpesvirus Vectored Vaccines

It has long been recognized that various vector-based vaccines have fundamental drawbacks, arising either from the nature of the vector itself, such as genetic instability, residual virulence, risk of genomic integration, and high seroprevalence within the population, or from technical feasibility issues, including sophisticated and expensive production processes that restrict the feasibility of large-scale manufacturing [77]. Herpesviral vectors inherently bear some of these limitations, which must be evaluated in advance for rational vectored vaccine design.

BoHV-1 is prevalent worldwide; for instance, a retrospective meta-analysis conducted in China revealed seropositivity rates of 43 percent in heifers and 46 percent in adult cattle, while the rate decreased to 14 percent in calves [78]. Substantial herpesvirus-acquired immunity does exist within the bovine population, and the protection of newborn calves strictly relies on passive immunisation via maternal antibodies [79]. Colostrum includes varied amounts of these antibodies, with a half-life of approximately 20–30 days for anti-BoHV-1, which could variably be detectable in calves between 2–10 months, depending on the methods applied [80,81,82]. Maternally derived antibody against BoHV-1 markedly diminishes vaccination efficacy and, even more critically, does not provide sufficient protection against natural infection [80,82,83]. Therefore, it is necessary to develop strategies capable of inducing an immune response “in the face of maternal antibody” (IFOMA). Furthermore, commercial vaccines largely fail to prevent reactivation by wild-type BoHVs in vaccinated animals. For instance, Petrini et al. (2022) reported that although gE-deleted vaccines elicit a strong immune response to protect against wild-type infection, they do not prevent latent viral reactivation, except for gE^−^/tk^−^ construct, which might confer partial protection [84]. A similar study by Zhang et al. (2011), however, demonstrated that reactivation was absent in both calves and rabbits IN-vaccinated with the gG^−^/tk^−^ double-knockout virus [85]. Taken together, using a multiple gene-knockout strategy with appropriate gene combinations may be a promising approach for generating herpesviral vectors that mitigate reactivation-related diseases.

Herpesviruses infect the ganglion and lymphoid tissues and establish lifelong latency via latency-associated transcripts (LATs); several factors (e.g., stress or using glucocorticoids) may eventually reactivate the virus and reignite active infection [19,86,87,88,89]. Thus, an emerging concern for herpesvirus vectors is that they may remain latent in vaccinated animals and, more critically, periodically shed virus. For instance, a study by Hill et al. (2019) demonstrated that calves vaccinated intranasally with a modified live vaccine at 3–6 weeks of age were still unable to prevent virus shedding when later challenged with a wild-type BoHV-1 strain; however, administering an IN booster at 6 months of age significantly reduced viral shedding [90]. Furthermore, a recent study by Pavulraj et al. (2024) investigated whether a quadruple-deleted, RWFV-Gn/GC/GMCSF–bearing bivalent vaccine candidate [26] established latency, could reactivate upon induction, and shed virus. Their results showed that both the vector virus control (Gc-null) and the bivalent virus established dexamethasone-inducible latency in the trigeminal ganglia; however, despite strong expression of LAT, ICP0, and gC in the ganglia, anterograde transport did not occur, and no virus shedding was detected in vaccinated animals [57]. Overall, results have been promising in terms of balancing safety and efficacy, but they have yet to be tested under field conditions.

Potential shortcomings of bovine herpesvirus-vectored vaccine production arise from several technical factors as well. First, recombinant vaccines are classified as GMOs, necessitating comprehensive risk assess potential hazards to human and environmental health [91]. Furthermore, each viral vector requires its own dedicated cell culture–based production system, which in turn demands investment in manufacturing facilities tailored to that specific platform [92]. It is plausible to anticipate that production will require animal-derived components such as cell lines, enzymes, and sera; therefore, a well-optimized downstream purification process and strict quality control measures are essential to mitigate the risk of contaminating pathogens. Notwithstanding, bovine herpesviruses exhibit a strong capacity to adapt to various culturing systems, ranging from broad-host-range cell lines to embryonated eggs [93,94,95,96]. This versatility offers significant advantages for herpesviral vector platforms, enabling scalable production across diverse manufacturing systems. BoHV-1 vaccines are conventionally produced using adherent BT or MDBK cells; yet only MDBK cells have been successfully adapted to serum-free suspension systems suitable for large-scale production [97] and already patented [98], whereas attempts to adapt BT cells have been unsuccessful [99].

As extensively exemplified in Table 1, despite the prevalent utilisation of BoHV-4 for the expression of heterologous genes, and although the majority of attempts have yielded promising results in in vivo studies, there are no commercially available vaccines. In 2012, a bacterial artificial chromosome (BAC)–based strategy for the production of BoHV-4 mutants expressing foreign genes for use as vaccine vectors was patented [100] by Vanderplasschen et al. (2012), who identified a major shortcoming of BoHV-4 as existence of ORF73 gene could result in latency, therefore excised from their engineered virus. The limited translation of BoHV-4–vectored vaccines into commercial products is likely attributable, in part, to regulatory barriers and the lack of a standardised manufacturing pipeline.

## 7. Conclusions and Perspectives

Although no recombinant BoHV-vectored vaccine has yet been commercialised, numerous studies have demonstrated that the genomic organisation of bovine herpesviruses provides a robust molecular framework for their development as viral vectors capable of delivering heterologous antigens. Furthermore, the commercial availability of gE-knockout vaccines (e.g., Tracherine^®^, Zoetis, Leatherhead, UK Limited; Bovilis^®^ IBR Marker, MSD Animal Health Inc., Rahway, New Jersey, USA) and gE/tk double-knockout vaccines (Hiprabovis^®^, Hipra Animal Health Inc., Amer (Girona), Spain) underscore the versatility of bovine herpesviruses and their suitability for scalable biomanufacturing. In this regard, molecular approaches such as homologous recombination, BAC technology, and CRISPR facilitate relatively easy and precise genetic editing of viral vectors, including the deletion of multiple genes. Multigene deletion would produce live-attenuated vector vaccines that are safe, immunogenic, and less prone to viral spread, thereby reducing the safety risks typically associated with live vaccines. In addition, the broad species tropism of BoHV-based vectors enables the development of vaccines targeting diverse viral diseases across multiple host species, thereby enhancing their protective applicability and advancing research into future zoonotic and livestock vaccine platforms. BoHV-vectored vaccine candidates may align well with a One Health strategy to prevent disease through mass vaccination and contribute substantially to control and prevention efforts. Thus, continued research should prioritise vaccine efficacy trials, dose optimisation, and comprehensive immunogenicity and safety assessments in larger and more diverse cattle populations to strengthen the evidence base for field use. Moreover, further investigation will be required to explore and validate vector vaccine candidates through the application of evolving molecular approaches.

## Data Availability

No new data were created or analyzed in this study. Data sharing is not applicable to this article.

## Abbreviations

The following abbreviations are used in this manuscript:

Ab	Antibody
BAC	Bacterial artificial chromosome
BRSV	Bovine respiratory syncytial virus
BSL-2	Biosecurity level 2
BVDV	Bovine viral diarrhea virus
CCHFV	Crimean-Congo hemorrhagic fever virus
dpv	Day post-vaccination
FMDV	Foot-and-mouth disease virus
GMO	Genetically modified organism
GMCSF	Granulocyte-macrophage colony-stimulating factor
HR	Homologous recombination
IFOMA	In the face of maternal antibody
IN	Intranasal
IM	Intramuscular
LAT	latency-associated transcripts
NA	Neutralising antibody
PPRV	Peste des petits ruminants virus
RE	Restriction enzyme
RVFV	Rift Valley fever virus
SPF	specific pathogen-free
TK	Thymidine kinase
